# How to conduct research on overdiagnosis. A keynote paper from the EGPRN May 2016, Tel Aviv: ‘Life can only be understood backwards; but it must be lived forwards’ Søren Kierkegaard (Danish philosopher 1813–1855)

**DOI:** 10.1080/13814788.2017.1290795

**Published:** 2017-03-16

**Authors:** John Brodersen

**Affiliations:** ^a^ Section of General Practice, Department of Public Health, Faculty of Health Sciences, University of CopenhagenDenmark; ^b^ Research Unit for General Practice, Department of Public Health, Faculty of Health Sciences, University of CopenhagenDenmark; ^c^ Primary Health Care Research Unit, Region ZealandDenmark

**Keywords:** Overdiagnosis, overuse, overtreatment, qualitative research, quantitative research

## Abstract

Overdiagnosis is a growing problem worldwide. Overdiagnosis is the diagnosis of deviations, abnormalities, risk factors, and pathologies that in themselves would never cause symptoms (this applies only to risk factors and pathology), would never lead to morbidity, and would never be the cause of death. Overdiagnosis is often misinterpreted as overutilization or overtreatment. Overutilization, overtreatment, and overdiagnosis are interrelated but three distinct topics. Overutilization (establishment of standard practice that does not provide net benefit) does not have to lead to overdiagnosis or overtreatment, but the risk exists. Treatment of overdiagnosed conditions is one category of overtreatment. Another is when the best available evidence shows that the treatment has no beneficial effect. Overdiagnosis can be caused by overutilization and is nearly always followed by overtreatment. Treating an overdiagnosed condition cannot improve the patient’s prognosis, and therefore can only be harmful. At the individual level, we can never be sure if the person is overdiagnosed. However, experiences and thoughts of individuals who are most likely overdiagnosed can be explored in qualitative interviews, e.g. men with a small screening detected abdominal aortic aneurism. In longitudinal surveys, the degree and length of psychosocial consequences associated with overdiagnosis can be estimated. In high-quality RCTs, the magnitude of overdiagnosis can be quantified, and in cohort studies, we can find indications of overdiagnosis. Finally, we can conduct research about the consequences of overdiagnosis in at least eight different areas: financial strain, hassles/inconveniences, medical costs, opportunity costs, physical harms, psychological harms, societal costs and work-related costs.

KEY MESSAGES

- The greatest methodological challenges associated with research on overdiagnosis are that at the individual level, we can never be sure if the person is overdiagnosed.
- If different methods are used it is possible to investigate different aspects of overdiagnosis, e.g. the experiences, the magnitude and the consequences of overdiagnosis.

## Introduction

The EGPRN arranges biannually a two-day conference targeting a specific theme. At the EGPRN May 2016 conference in Tel Aviv, the theme was ‘Research on medical overuse: Overdiagnosis and overtreatment in family medicine and primary care.’ Overdiagnosis is a growing problem all over the world, especially in high-income countries, because of three reasons: (1) overdetection due to more sensitive tests, more testing, more screening and earlier diagnosis; (2) widening disease definitions and lowering of thresholds and; (3) disease mongering [1]. This paper encompasses the themes from a keynote lecture titled ‘How to conduct research on overdiagnosis’ presented at the EGPRN meeting in May 2016. Different topics and methodological issues concerning conducting research on overdiagnosis are presented and discussed. First, overdiagnosis is defined and the dilemmas and pitfalls in all diagnostic processes in a general practitioner’s (GP’s) clinical practice are described. Second, suggestions of how to conduct research in overdiagnosis qualitatively and quantitatively are revealed. Finally, implications for future research are put in perspective.

## Definition of overdiagnosis

Overdiagnosis is the diagnosis of deviations, abnormalities, risk factors, and pathologies that in themselves would never cause symptoms (this applies only to risk factors and pathology), would never lead to morbidity, and would never be the cause of death. Treating an overdiagnosed condition (deviation, abnormality, risk factor, and pathology) cannot, by definition, improve the patient’s prognosis, and therefore can only be harmful [1–3]. Treatment of overdiagnosed conditions is one category of overtreatment. Another type of overtreatment is when the best available scientific evidence shows that the treatment has no beneficial effect on the diagnosed condition and may even be harmful [4]. Overuse, or better described as overutilization, is the ‘establishment of standard practice in health services or systems that do not provide net benefit to patients or citizens’ [4]. Overutilization does not necessarily lead to overdiagnosis or overtreatment, but the risk increases proportionally with the degree of overutilization.

## The dilemmas and pitfalls of the diagnostic process in general practice

We can never, as GPs (or patients for that matter), be certain at the level of the individual patient whether he or she has in fact been overdiagnosed. Only at the end of the patient’s life can we for biomedical conditions confirm whether our diagnosis was correct or iatrogenic.

For psychosocial conditions and illnesses, or mental disorders, we can never answer that question conclusively. Therefore, the dilemmas and pitfalls in all diagnostic processes in a GP’s daily clinical patient-centred practice—with low prevalence of biomedical diseases and high prevalence of psychosocial illnesses—is so succinctly captured by this quote from the Danish philosopher Søren Kirkegaard (1813–1855): ‘Life can only be understood backwards; but it must be lived forwards.’ Hence, the million dollars (or more accurately multi-billion dollars) question: how to conduct research about something that cannot be directly observed (overdiagnosis), and yet attempt to reduce or prevent its prevalence?

## How to conduct research on overdiagnosis

### Qualitative studies

If we want to know more about ‘lived life’ (e.g., the experiences and thoughts of individuals that have been overdiagnosed), we will most likely raise research questions that can be addressed in qualitative designs, such as interviews and observational fieldwork. However, because we can never be certain that the individual has been overdiagnosed, we can interview those informants, who are most likely to be overdiagnosed, or informants that for a short period have had the experience of being overdiagnosed (false positives).

For example, Reventlow et al. interviewed 16 healthy women with no chronic or disabling conditions, and who had been (over)diagnosed with osteoporosis via a population-based cohort study [5]. The interviews revealed that the participating women appeared to take the scan literally. They planned their lives accordingly, believed that the ‘pictures’ revealed some truth, and interpreted the scan result to mean real fragility, which they incorporated into their real perception.

Hansson et al. interviewed 15 men who via screening were (over)diagnosed with an abdominal aortic aneurism (AAA) with a median aorta-diameter of 32 mm [6]. The men expressed ambivalence towards the diagnosis: ‘they appreciated having the knowledge but it was accompanied by worry, feelings of anxiety and existential thoughts about the fragility and finiteness of life.’

Brodersen et al. interviewed in a focus group lung cancer screening participants, who had an abnormal thorax CT scan that later was confirmed to be false positive [7]. The critical period from when the abnormal screening result was acknowledged to the point in time the screening participant was cleared of suspicion of lung cancer was three months or more. These screening participants reported substantial negative psychosocial consequences from living with the uncertainty of possibly having lung cancer.

### Survey research

We can also conduct longitudinal studies using condition-specific questionnaires that encompass items developed from the qualitative work described above if we want to address research questions about the degree to which women are anxious after a false-positive screening mammography, or how long this anxiety lasts [8]. A survey using a condition-specific questionnaire for women who had participated in screening mammography, which included more than 1300 women, revealed that the women still reported substantial negative psychosocial consequences three years after the false-positive screening mammography [9].

### Quantitative studies

*Estimating the degree of overdiagnosis by cumulative incidence.* If we are not only interested in the how and what individuals experience when they have been overdiagnosed but want to ascertain the number of people overdiagnosed, then quantitative research designs are required. The best available scientific evidence is produced through high-quality randomized controlled trials (RCTs). The next best evidence comes from cohort studies. The least convincing evidence is results generated from simulation studies and other statistical modelling studies not validated with empirical data [10].

The simplest and most robust way of estimating the degree of overdiagnosis in a screening RCT would be from the start of screening trial to estimate the cumulative incidence of the condition screened for in the control group and the intervention group. However, two important types of biases are in play, biases that are normally not included in the standard bias assessment tools of RCTs. The first is lead-time bias: the two groups must be followed for a sufficient time-period after the end of screening. A too short follow-up can otherwise overestimate the degree of overdiagnosis. In the European randomized study of screening for prostate cancer, it has for example been shown that after the nine-year follow-up the ratio between one prevented death from prostate cancer and men overdiagnosed with prostate cancer were 1:47, while this ratio decrease to 1:37 at the 11-year follow-up and 1:27 after 13 years of follow-up [11–13]. The second is contamination bias: if participants in the control group are either opportunistically screened or are having some diagnostic work-up for the condition screened for, then the degree of overdiagnosis can be underestimated [14]. In the prostate, lung, colorectal, and ovarian (PLCO) cancer screening trial from the US more than 80% of the men randomized to the control group in the PSA-screening part of the trial reported having undergone at least one PSA test [15]. The degree of overdiagnosis of prostate cancer in the PLCO trial was reported to be only 21% while other RCTs on PSA screening where the control group were less contaminated with PSA testing reported degrees of overdiagnosis of 50–63% [16].

In most screening trials and ongoing screening programmes where the target of the screening is the actual disease and not precursors, the yearly incidence of the clinically manifest disease screened for is around 0.5% and overdiagnosis varies from 10% to more than 50% depending on the disease screened for and what screening test is used. Moreover, the mortality reduction is around 15–30%, again depending on the disease screened for and which screening test is used. Thus, small absolute numbers and effect sizes are in play. This means that even a minor bias in an RCT can affect these numbers substantially. This is for example didactically illustrated in the Cochrane review for screening mammography, where a meta-analysis including nine RCTs shows a breast cancer mortality after a 13-year follow-up to be 0.81 (0.74, 0.87) [17]. However, if only the four adequately randomized trials were included in the meta-analysis, the estimate was 0.90 (0.79, 1.02) while the estimate of the five remaining insufficiently randomized trials were 0.75 (0.67, 0.83) [17]. Hence, including all nine RCTs shows a 19% statistically significant relative mortality reduction in breast cancer, a statistically significant 25% mortality when only the biased trials are included while including only the non-biased trails shows a non-statistically significant reduction of 10%. This illustrates why the need for high-quality RCTs in medical screening is essential if results on benefits and harms are to be trusted. However, in most cancer screening RCTs data on overdiagnosis are not published [18]. Therefore, when conducting randomized controlled screening trials the focus should be not only on high-quality study design minimizing biases but also on investigating both the potential intended positive effects and the potential unintended negative effects of screening.

## Stage shift

Another possible outcome to measure in screening trials that can indicate the degree of overdiagnosis is stage-shift: a shift to earlier stage at diagnosis. However, while a relative stage-shift does not tell us if the screening is benefitting or harming, an absolute stage-shift is easier to interpret. Esserman and colleagues describe in their paper ‘Rethinking screening for breast cancer and prostate cancer’ three screening scenarios: optimal, worst case, and intermediate [19]. The didactical clue in Esserman’s paper is that while the relative stage-shifts in the three scenarios are the same, the absolute stage-shifts are very different [19]. An empirical example of this is the Danish lung cancer screening trial (DLCST). At the end of five yearly screening rounds, a relative, but not an absolute, stage-shift was revealed [20]. After five years of follow-up of the DLCST, the same results were confirmed, indicating a considerable degree of overdiagnosis in lung CT screening [21].

## Standardized incidence and standardized mortality

Finally, the trajectory of the standardized incidence and the standardized mortality of conditions screened for should be considered, including a time before and during a screening era. If the standardized incidence of the condition screened for increases after the start of screening and the standardized mortality does not change (and treatment of the condition in the same period has not improved substantially), this could indicate some degree of overdiagnosis [22]. The magnitude of overdiagnosis depends on how much the incidence of the condition screened for increases. Moreover, if this increased incidence continues to increase during the period of screening, or stays consistently elevated at the same increased level after the end of screening, there is robust evidence of overdiagnosis. However, if the increased incidence gradually stabilizes during screening or the incidence drops after the period of screening has ended, this is an indication of a compensatory drop in incidence due to lead-time. There is no evidence of overdiagnosis if the compensatory drop after the end of the screening is just as great as the increased incidence during the screening period. Furthermore, when screening for precursors, the compensatory drop in incidence can be even greater than the increased incidence during screening, which then is evidence of a primary preventive effect of the screening programme [23].

## How to calculate the magnitude of overdiagnosis

The magnitude of overdiagnosis can be estimated in several ways primarily because different denominators can be used [24]. Therefore, to compare two or more estimates of overdiagnosis, it is important to ensure that the estimates are actually calculating the degree of overdiagnosis using the same methodology. All health professionals, politicians, health authorities, patients, and citizens, in general, have a stake in the answer to the question: what is the risk of being overdiagnosed? However, to answer this question, the denominator or the comparator must be defined. The risk of being overdiagnosed in cancer screening could be split into numerous questions, for example, (1) how many in a cohort invited to screening are overdiagnosed with cancer? (2) How many of the screening participants are overdiagnosed with cancer? (3) How many of the screening-detected cancers are overdiagnosed? (4) How many deaths from cancer are prevented compared to how many screening participants are overdiagnosed with cancer? The list of questions can continue. In addition, the answers to these questions can be communicated in different ways (e.g., as percentages, percentage points, in absolute numbers, in odds, etc.). Research about how best to communicate overdiagnosis is in its early stages and more research is needed [25].

## The consequences of overdiagnosis

A final type of research question can focus on the consequences of overdiagnosis. Harris and colleagues have suggested a taxonomy describing seven different categories that could be explored: financial strain, hassles/inconveniences, medical costs, opportunity costs, physical harms, psychological harms, and societal costs [26]. In addition, we have identified empirical evidence for an additional category: work-related costs [27]. Both qualitative and quantitative study designs are needed to explore the empirical evidence in these eight categories of consequences of overdiagnosis.

## Implications for research

Overdiagnosis is an extremely harmful and sizeable problem all over the world and the problem is increasing. This is especially the case in high-income countries where more sensitive tests, more testing, more screening and earlier diagnosis occurs and more of the same will be implemented in the future. Moreover, disease definitions have been and are still being widened; plus thresholds for treating risk factors, for example, have been and are still being lowered. Finally, disease mongering is growing because it is cheaper and faster to invent new ‘diseases’ than new pharmaceutical drugs. In all aspects of overdiagnosis there is a substantial absence of evidence. Therefore, research is needed into the degree of overdiagnosis; the harm caused by overdiagnosis; the consequences of overdiagnosis; how to prevent overdiagnosis; and how to communicate overdiagnosis to physicians, other healthcare professionals, politicians, healthcare providers and stakeholders, and most importantly the general population.
